# Folate-related gene variants in Irish families affected by neural tube defects

**DOI:** 10.3389/fgene.2013.00223

**Published:** 2013-11-06

**Authors:** Ridgely Fisk Green, Julianne Byrne, Krista S. Crider, Margaret Gallagher, Deborah Koontz, Robert J. Berry

**Affiliations:** ^1^Carter Consulting, Inc. and Division of Birth Defects and Developmental Disabilities, National Center on Birth Defects and Developmental Disabilities, Centers for Disease Control and PreventionAtlanta, GA, USA; ^2^Boyne Research InstituteDrogheda, Ireland; ^3^Division of Birth Defects and Developmental Disabilities, National Center on Birth Defects and Developmental Disabilities, Centers for Disease Control and PreventionAtlanta, GA, USA; ^4^Newborn Screening and Molecular Biology Branch, Division of Laboratory Science, National Center for Environmental Health, Centers for Disease Control and PreventionAtlanta, GA, USA

**Keywords:** neural tube defects, folate metabolism, *DHFR* 19bp deletion, *MTHFD1* 1958G>A, *MTHFR* 1298A>C, *MTHFR* 677C>T, *SLC19A1* 80A>G, maternal inheritance

## Abstract

Periconceptional folic acid use can often prevent neural tube defects (NTDs). Variants of genes involved in folate metabolism in mothers and children have been associated with occurrence of NTDs. We identified Irish families with individuals affected by neural tube defects. In these families, we observed that neural tube defects and birth defects overall occurred at a higher rate in the maternal lineage compared with the paternal lineage. The goal of this study was to look for evidence for genetic effects that could explain the discrepancy in the occurrence of these birth defects in the maternal vs. paternal lineage. We genotyped blood samples from 322 individuals from NTD-affected Irish families, identified through their membership in spina bifida associations. We looked for differences in distribution in maternal vs. paternal lineages of five genetic polymorphisms: the *DHFR* 19 bp deletion, *MTHFD1* 1958G>A, *MTHFR* 1298A>C, *MTHFR* 677C>T, and *SLC19A1* 80A>G. In addition to looking at genotypes individually, we determined the number of genotypes associated with decreased folate metabolism in each relative (“risk genotypes”) and compared the distribution of these genotypes in maternal vs. paternal relatives. Overall, maternal relatives had a higher number of genotypes associated with lower folate metabolism than paternal relatives (*p* = 0.017). We expected that relatives would share the same risk genotype as the individuals with NTDs and/or their mothers. However, we observed that maternal relatives had an over-abundance of any risk genotype, rather than one specific genotype. The observed genetic effects suggest an epigenetic mechanism in which decreased folate metabolism results in epigenetic alterations related to the increased rate of NTDs and other birth defects seen in the maternal lineage. Future studies on the etiology of NTDs and other birth defects could benefit from including multigenerational extended families, in order to explore potential epigenetic mechanisms.

## Introduction

Neural tube defects (NTDs) are congenital anomalies of the brain and spinal cord that cause significant morbidity and mortality (Sutton et al., 2008). Historically, Ireland has had high rates of NTDs (Coffey, 1983), with rates decreasing significantly in the last 50 years (Botto et al., 2006). Both environmental and genetic factors have been implicated in the etiology of NTDs. The evidence for environmental factors lies in the protective effect of periconceptional folic acid intake (Czeizel and Dudas, 1992). The evidence for genetic factors lies in the increased recurrence risk in siblings of individuals with NTDs, the increased risk for NTDs among other relatives, and the increased risk for birth defects overall among both close and distant relatives (MRC Vitamin Study Research Group, 1991; Deak et al., 2008). Maternal relatives have higher NTD and overall birth defect rates than paternal relatives, consistent with a combination of genetic and epigenetic mechanisms contributing to NTD and overall birth defects risk (Byrne et al., 1996; Byrne, 2008, 2010, 2011; Deak et al., 2008).

The genetic mechanisms associated with NTDs have been addressed through studies on associations between NTD risk and variants in genes involved in folate metabolism, chosen because of the protective effect of folic acid on NTDs (Molloy et al., 2009; Shaw et al., 2009). Methylene tetrahydrofolate reductase (*MTHFR*) plays an important role in folate metabolism. The 677 T allele results in reduced enzyme activity and decreased folate metabolism (Frosst et al., 1995). The *MTHFR* 677 TT genotype in infants and mothers has been associated with increased risk for NTDs [reviewed in Botto and Yang (2000)]. Studies in Irish populations found significantly more individuals with NTDs with the TT and CT genotypes and more parents of affected individuals with the TT genotype (Whitehead et al., 1995; Shields et al., 1999; Kirke et al., 2004; Carter et al., 2011). The *MTHFR* 1298A>C variant also results in reduced enzyme activity, although its role in NTD risk is less clear (van der Put et al., 1998; Botto and Yang, 2000; Wang et al., 2012a). Parle-McDermott et al. (2003) did not find an association between the *MTHFR* 1298A>C variant and NTDs in an Irish population. Methylene tetrahydrofolate dehydrogenase 1 (*MTHFD1*) acts in folate metabolism and DNA synthesis, and the 1958G>A variant results in decreased enzyme stability (Christensen et al., 2009). Studies in Irish populations (Brody et al., 2002; Parle-McDermott et al., 2006) identified a significant excess of *MTHFD1* AA homozygotes among mothers of NTD cases compared to controls. A recent larger scale study in an Irish population also identified the *MTHFD1* A allele as a risk factor for NTDs (Pangilinan et al., 2012). Dihydrofolate reductase (*DHFR*) also acts in folate metabolism. Studies have yielded conflicting results on the effect of the 19 bp deletion at intron 1 on red blood cell folate concentrations (Kalmbach et al., 2008; Stanislawska-Sachadyn et al., 2008). Researchers from Trinity College in Dublin observed a slight protective effect of the homozygous *DHFR* deletion genotype in mothers (Parle-McDermott et al., 2007). *SLC19A1* plays an important role in folate transport, and some previous studies have reported associations between the 80A>G variant and NTD risk (Chango et al., 2000; De Marco et al., 2003; Wang et al., 2012b). O'Leary et al. (2006) did not find a difference in the frequency of the *SLC19A1* 80A>G reduced folate carrier in mothers, fathers and affected individuals compared with controls in an Irish population.

Epigenetic mechanisms related to NTDs have not been well explored. The higher rates of NTDs in maternal relatives compared with paternal relatives suggest the presence of epigenetic effects. Folate metabolism genes are of interest because folate metabolism is critical to the production of one-carbon subunits needed for epigenetic modification (Crider et al., 2012). Decreased folate metabolism, seen with certain gene variants, might hinder proper establishment or maintenance of epigenetic modifications. The epigenetic changes of interest would occur in the maternal genome *in utero*, that is, during the grandmother's pregnancy (Crider et al., 2012). Thus, in contrast to genetic mechanisms, in which the mothers' and affected individuals' genotypes would be most relevant, for epigenetic mechanisms, the maternal and paternal grandparents' genotypes would be important. In the absence of information on grandparents' genotypes, aunts, uncles, and first cousins provide an indirect measure of the grandparents' genotypes.

In this hypothesis-generating study, we investigated possible mechanisms for the excess risk for NTDs and birth defects overall in the maternal lineage of Irish individuals with NTDs. To do this, we evaluated five polymorphisms in genes involved in folate metabolism in the maternal vs. paternal lineage, both individually and with low folate-metabolizing genotypes grouped together. To our knowledge, this study is the first to look for genomic associations in extended relatives in NTD families and the effect of maternal vs. paternal lineage risk. We chose five polymorphic markers for evaluation, which were among the most intensely studied so far: the 19 bp deletion in *DHFR*, *MTHFD1* 1958G>A, *MTHFR* 1298A>C, *MTHFR* 677C>T, and *SLC19A1* 80A>G.

## Materials and methods

### Study design and participants

The participants in this cross-sectional study were members of Irish families with NTDs. These families had previously participated in epidemiologic studies of the Boyne Research Institute [for instance, (Byrne, 2011)]. The families were identified initially through their membership in the Louth-Meath (northeastern region of the Republic of Ireland) branch of the Irish Association for Spina Bifida and Hydrocephalus, and the Ballymena (Northern Ireland) branch of the (UK) Association for Spina Bifida and Hydrocephalus. Families were also recruited through word of mouth and through radio announcements in the Louth-Meath area.

To be eligible for the study, male and female participants had to be 15 years of age or older, reside in the northeast region of the Republic of Ireland or Northern Ireland, and be either the individual with an NTD (only one NTD in sibship) or a blood relative of the affected individual. In terms of relationship, relatives (defined according to their relationship to the affected individual) had to be one of the following: parents, siblings, nieces, nephews, offspring (of the affected individual), grandparents, uncles, aunts, first cousins and first cousins once removed (the offspring of the first cousins).

Family members were interviewed in phases. Phase I evaluated pregnancy histories and the health of siblings in the original nuclear families (1995–2002). Phase II subjects (uncles and aunts of the affected individuals) were identified by their siblings during Phase I and interviewed between 2000 and 2002. Phase III consisted of interviews with first cousins of the affected individuals (the children of the uncles and aunts) between 2002 and 2009. Between 2005 and 2006, affected individuals and their siblings were interviewed.

For the present study, conducted in 2007, the response rate among eligible, locatable relatives was 49.5%, or 331 participants. From these, 322 gave a blood sample. Of these with a blood sample, 34 were relatives in families in which the individual with an NTD had anencephaly, 41 in which the affected individual had spina bifida occulta, 240 in which the affected individual had open spina bifida and 7 in which the affected individual had encephalocele.

This study was conducted according to the guidelines from the Declaration of Helsinki and all procedures involving human subjects were approved by the Ethics Board of the Boyne Research Institute and the Ethics Committee of the Health Service Executive of the Irish Government's Department of Health and Children. Written informed consent was obtained from all subjects.

### Statistical analysis

Data were managed using Microsoft EXCEL^©^, and statistical analysis was conducted using SAS 9.1 (SAS Institute, Cary, NC, USA), STATA^©^, and IBM® SPSS® v19. Measures of association were evaluated with *t*-tests for continuous variables and chi-square and Fisher's exact tests for categorical variables, with the level of statistical significance set at 0.05. First-degree relatives were parents, siblings, and children; second-degree relatives were aunts, uncle, nieces, nephews, and grandparents; and third-degree and higher relatives were cousins and first cousins once removed. Genotypes were analyzed using an additive model, consistent with previous studies. For logistic regression analyses, genotypes were dichotomized using a recessive model, consistent with previous studies. For each individual, we calculated the number of “risk genotypes” present. “Risk genotypes” were those associated with lower folate metabolism in previous studies (*DHFR* WT/WT, *MTHFD1* 1958 AA, *MTHFR* 1298 CC, *MTHFR* 677 TT, and *SLC19A1* 80 GG).

### DNA methods

DNA was extracted from blood clots using Gentra Puregene Blood kit and Clotspin Baskets per manufacturer's protocol (Qiagen, Valencia, CA). Quantitation was performed by spectrophotometric reading at 260nm and all samples normalized to 10 ng/μl. For PCR reactions, 2 μl genomic DNA (20 ng) was used. Pyrosequencing technology (Qiagen, Valencia, CA) was used to genotype DNA samples for *DHFR* 19 bp deletion (rs70991108), *MTHFD1* 1958G>A (rs2236225), *MTHFR* 1298A>C (rs1801131), *MTHFR* 677C>T (rs1801133), and *SLC19A1* 80A>G (rs1051266). PCR and pyrosequencing primers (Table 1) were designed using PSQ Assay Design Software Version 1.0.6. After amplification, the pyrosequencing primer was used to extend the PCR product by addition of nucleotides in a specific sequential order based on the known sequence. The resulting pyrogram of signal peaks was used to determine the genotype. PCR amplification for the *DHFR* 19 bp deletion was performed using the GC Rich PCR System (Roche, Indianapolis, IN) as previously described (Parle-McDermott et al., 2007). PCR for all other variants was performed in a 20 μl reaction mix containing 0.5 μM each forward and reverse primer and Applied Biosystems GeneAmp® Fast PCR Master Mix. Amplification was performed using the 9800 Fast PCR System according to manufacturer's suggested protocol (Applied Biosystems, Foster City, CA). Pyrosequencing was carried out as described by manufacturer's protocol (Qiagen, Valencia, CA).

**Table 1:** **Primer sequences and PCR amplicon size for *DHFR*, *MTHFD1*, *MTHFR*, and *SLC19A1* variant analysis**.

**Variant**	**Forward primer (5′–3′)^a^**	**Reverse primer (5′–3′)^a^**	**Sequencing primer (5′–3′)^b^**	**Amplicon size (bp)**
*DHFR* 19 bp deletion (rs70991108)	CAAGAACGGGGACCTGCC	^*^ATCCTCTCGCCGGGAGTC	GCCCACGGTCGGGGT	114,133
*MTHFD1* 1958G>A (rs2236225)	TCATTTTGCTTAGAGCACAGTAGA	^*^CATCCACTTTCCTGGCTTAAAT	CACAGTAGAGAGTGCCAA	63
*MTHFR* 1298A>C (rs1801131)	GGAGCTGCTGAAGATGTGG	^*^TGGTTCTCCCGAGAGGTAAAG	AGGAGCTGACCAGTGA	85
*MTHFR* 677C>T (rs1801133)	^*^ATTGGCAGGTTACCCCAAAGG	TCGGTGCATGCCTTCACAA	CGTGATGATGAAATCG	156
*SLC19A1* 80A>G (rs1051266)	^*^GTGGAACCTGGGCCTGAC	GTAGGGGGTGATGAAGCTCTC	CAAAGGTAGCACACGA	114

a* *Biotinylated at 5′ end*.

b *Represent forward primers for DHFR, MTHFD1, MTHFR 1298, and reverse primers for MTHFR 677 and SLC19A1*.

*^c^MTHFR 677 and SLC19A1 assays on reverse complement strand*.

### Quality control

Five percent of the samples were selected at random for repeat testing of the five polymorphisms, and the concordance was checked. We also tested each variant for any significant departure from Hardy-Weinberg equilibrium (HWE) by a chi-square goodness-of-fit test to verify data quality. In addition, Mendelian inheritance for all variants was assessed in 13 families with full trios using the PedCheck program (O'Connell and Weeks, 1998). Water and genotype controls (Coriell Cell Repositories, Camden, NJ) were included on each assay plate. The tests for HWE indicated that none of the variants deviated significantly from HWE, supporting high quality genotype data. All replica test results were 100% concordant.

## Results

We obtained blood samples from all categories of relatives, including individuals with NTDs (*n* = 13), offspring (*N* = 2), fathers (*N* = 29), mothers (*N* = 37), siblings (*N* = 59), nieces and nephews (*N* = 2), uncles and aunts (*N* = 66), first cousins (*N* = 105), and grandparents (*N* = 2) from 47 families. Paternal relatives (*N* = 65) and maternal relatives (*N* = 115) comprised uncles and aunts, first cousins, first cousins once removed, and grandparents, who were related to the affected individual through the father and mother, respectively. The average age of the study participants was 41 years. The overall distribution of each of the five polymorphisms is shown in Table 2. The genotype distributions for the five polymorphisms in our study were similar to those from other studies, with a few exceptions (Table 3).

**Table 2:** **Characteristics of the participants, northeast region of the Republic of Ireland and Northern Ireland, 1995–2009**.

		**Range**	**Median**
Age in years	Overall	16–86	41.5
	Affected individual	18–47	28.8
	1st degree	17–73	44.7
	2nd degree	18–86	52.1
	3rd degree or higher	16–59	33.5
		***N***	**%**
Gender	Males	120	37.3
	Females	202	62.7
Relationship	Affected individual	13	4.0
	Offspring (of affected individual)	2	0.6
	Father	29	9.0
	Mother	37	11.5
	Sibling	59	18.3
	Niece/Nephew	2	0.6
	Uncle/aunt	66	20.5
	First cousin	105	32.6
	First cousin once removed	7	2.2
	Grandfather	1	0.3
	Grandmother	1	0.3
	1st degree	127	39.4
	2nd degree	70	21.7
	3rd degree or higher	112	34.8
Genotyping results	*DHFR* 19 bp deletion		
	del/del	61	19.0
	WT/del	172	53.4
	WT/WT	89	27.6
	*MTHFD*1 1958G>A		
	AA	51	15.8
	AG	156	48.5
	GG	115	35.7
	*MTHFR* 1298A>C		
	AA	145	45.0
	AC	147	45.7
	CC	30	9.3
	*MTHFR* 677C>T		
	CC	134	41.6
	CT	156	48.5
	TT	32	9.9
	*SLC19A1* 80A>G		
	AA	58	18.0
	AG	168	52.2
	GG	96	29.8
Line	Paternal relatives	65	36.0
	Maternal relatives	115	64.0

**Table 3:** **Distribution of genotypes and comparison with other studies**.

***DHFR***		**Del/Del**		**Del/WT**		**WT/WT**		***P***
		***N***	**%**	***N***	**%**	***N***	**%**	
This study	Ireland, NTD relatives	61	18.9	172	53.5	89	27.6	Ref.
Parle-McDermott et al., 2007	Ireland, controls	46	18.6	128	51.8	73	29.6	0.88
Kalmbach et al., 2008	US, unaffected	216	17.1	646	51.4	396	31.5	0.39
This study	Ireland, mothers	7	18.9	22	59.5	8	21.6	Ref.
Parle-McDermott et al., 2007	Ireland, mothers	49	18.0	130	47.0	96	35.0	0.25
***MTHFD1***		**A/A**		**A/G**		**G/G**		
This study	Ireland, NTD relatives	51	15.7	156	48.6	115	35.7	Ref.
HapMap (CEU)	Utah	20	17.7	54	47.8	39	34.5	0.90
Parle-McDermott et al., 2006	Ireland, controls	150	19.5	411	53.4	209	27.1	0.02
This study	Ireland, Mothers	8	21.6	17	46.0	12	32.4	Ref.
Parle-McDermott et al., 2006	Ireland, mothers	65	27.0	118	48.0	62	25.0	0.62
***MTHFR* 1298**		**A/A**		**A/C**		**C/C**		
This study	Ireland, NTD relatives	145	45.0	147	45.7	30	9.3	Ref.
HapMap (CEU)	Utah	49	43.4	51	45.1	13	11.5	0.79
van der Put et al., 1998	Holland, controls	179	44.4	186	46.2	38	9.4	0.99
Mills et al., 2008	Ireland, controls	519	49.4	439	41.8	92	8.8	0.38
***MTHFR* 677**		**C/C**		**C/T**		**T/T**		
This study	Ireland, NTD relatives	134	41.6	156	48.6	32	9.8	Ref.
HapMap (CEU)	Utah	53	47.0	50	44.2	10	8.8	0.62
Shields et al., 1999	Ireland, controls	114	47.1	108	44.6	20	8.3	0.41
Mynett-Johnson et al., 2002	Ireland, unaffected volunteers	209	34.7	299	49.7	94	15.6	0.02
This study	Ireland, affected individuals	2	15.4	9	69.2	2	15.4	Ref.
Shields et al., 1999	Ireland, affected individuals	101	37.3	119	43.9	51	18.8	0.18
This study	Ireland, mothers	17	46.0	18	48.6	2	5.4	Ref.
Shields et al., 1999	Ireland, mothers	80	36.7	108	49.5	30	13.8	0.29
***SLC19A1***		**A/A**		**A/G**		**G/G**		
This study	Ireland, NTD relatives	58	18.0	168	52.2	96	29.8	Ref.
HapMap (CEU)	Utah	18	16.0	62	55.4	32	28.6	0.83
O'Leary et al., 2006	Ireland, controls	103	27.0	179	47.0	99	26.0	0.02
This study	Ireland, affected individuals	4	30.8	7	53.9	2	15.3	Ref.
O'Leary et al., 2006	Ireland, affected individuals	61	23.0	131	50.0	72	27.0	0.60
This study	Ireland, mothers	7	18.9	19	51.4	11	29.7	Ref.
O'Leary et al., 2006	Ireland, mothers	65	25.0	119	45.0	80	30.0	0.70
This study	Ireland, fathers	7	24.1	16	55.2	6	20.7	Ref.
O'Leary et al., 2006	Ireland, fathers	58	22.0	127	48.0	79	30.0	0.58

### Analysis of individual polymorphisms

The distributions of each genotype and allele by relationship to the individual with an NTD, degree of relationship to the affected individual, and maternal vs. paternal lineage are shown in Table 4. We did not observe any significant associations between individual genotypes and the affected individuals or mothers, although the higher folate-metabolizing *MTHFR* 677 CC genotype tended to be found less often in affected individuals (15.4%) than in all other relatives (42.7%) (Fisher's exact test, *p* = 0.08) (Table 4 and data not shown). We also did not see any significant associations between individual genotypes and maternal vs. paternal lineage (Table 4).

**Table 4:** **Distribution of genotypes and alleles according to relationship within NTD-affected families and lineage, northeast region of the Republic of Ireland and Northern Ireland, 1995–2009**.

***DHFR***	**Genotype frequencies**						***P*^b^**	**Allele frequencies**				***P*^b^**
	**Del/Del**		**Del/WT**		**WT/WT**			**Del**		**WT**		
	***N***	**%**	***N***	**%**	***N***	**%**		***N***	**%**	***N***	**%**	
Affected individual	2	15.4	6	46.2	5	38.4	0.74	10	38.5	16	61.5	0.45
Mother	7	18.9	22	59.5	8	21.6	0.66	36	48.6	38	51.4	0.58
Father	6	20.7	15	51.7	8	27.6	0.97	27	46.6	31	53.4	0.89
Sibling	13	22.0	35	59.3	11	18.7	0.23	61	51.7	57	48.3	0.13
Uncle/aunt	16	24.2	30	45.5	20	30.3	0.30	62	47.0	70	53.0	0.71
First cousin	13	**12.4**	61	58.1	31	29.5	**0.11**	87	41.4	123	58.6	0.13
Other rel.	4	30.8	3	23.0	6	46.2	0.06	11	42.3	15	57.7	0.73
1st degree relatives	26	20.5	72	56.7	29	22.8	0.36	124	48.8	130	51.1	0.23
2nd degree relatives	17	24.3	33	47.1	20	28.6	0.36	67	47.9	73	52.1	0.61
3rd degree or higher relatives	16	14.3	61	54.5	35	31.2	0.20	93	41.5	131	58.5	0.10
Paternal line^a^	9	13.8	37	56.9	19	29.3	0.40	55	42.3	75	57.7	0.65
Maternal line^a^	24	20.9	55	47.8	36	31.3		103	44.8	127	55.2	
***MTHFD1***	**G/G**		**G/A**		**A/A**			**G**		**A**		
Affected individual	2	15.4	8	61.5	3	23.1	0.24	12	46.2	14	53.8	0.14
Mother	12	32.4	17	46.0	8	21.6	0.59	41	55.5	33	44.6	0.40
Father	14	48.3	10	34.5	5	17.2	0.26	38	65.5	20	34.5	0.36
Sibling	16	27.2	32	54.2	11	18.6	0.31	64	54.2	54	45.8	0.16
Uncle/aunt	34	**51.5**	28	42.4	4	6.1	**0.003**	96	72.7	36	27.3	0.0008
First cousin	34	32.4	53	50.5	18	17.1	0.68	121	57.6	89	42.4	0.40
Other rel.	3	23.1	8	61.5	2	15.4	0.55	14	53.8	12	46.2	0.52
1st degree relatives	43	33.9	60	47.2	24	18.9	0.36	146	57.5	108	42.5	0.20
2nd degree relatives	35	50.0	30	42.9	5	7.1	0.01	100	71.4	40	28.6	0.003
3rd degree or higher relatives	35	31.2	58	51.8	19	17.0	0.34	128	55.4	96	42.9	0.20
Paternal line	23	35.4	36	55.4	6	9.2	0.24	82	63.1	48	36.9	0.93
Maternal line	47	40.9	50	43.5	18	15.6		144	62.6	86	37.4	
***MTHFR* 1298**	**A/A**		**A/C**		**C/C**			**A**		**C**		
Affected individual	6	46.2	6	46.2	1	7.6	1.00	18	69.2	8	30.8	0.88
Mother	15	40.5	17	46.0	5	13.5	0.61	47	63.5	27	36.5	0.40
Father	11	37.9	15	51.8	3	10.3	0.72	37	63.8	21	36.2	0.49
Sibling	18	**30.5**	35	59.3	6	10.2	**0.04**	71	60.2	47	39.8	0.05
Uncle/aunt	33	50.0	26	39.4	7	10.6	0.52	92	69.7	40	30.3	0.61
First cousin	54	51.4	44	41.9	7	6.7	0.22	152	72.4	58	27.6	0.09
Other rel.	8	61.5	4	30.8	1	7.7	0.49	20	76.9	6	23.1	0.31
1st degree relatives	44	**34.6**	68	53.6	15	11.8	**0.009**	156	**61.4**	98	38.6	**0.005**
2nd degree relatives	35	**50.0**	28	40.0	7	10.0	0.56	98	**70.0**	42	30.0	0.53
3rd degree or higher relatives	60	**53.6**	45	40.2	7	6.2	0.05	165	**73.7**	59	26.3	0.02
Paternal line	30	46.3	29	44.5	6	9.2	0.53	89	68.5	41	31.5	0.27
Maternal line	63	54.8	44	38.2	8	7.0		170	73.9	60	26.1	
***MTHFR* 677**	**C/C**		**C/T**		**T/T**			**C**		**T**		
Affected individual	2	**15.4**	9	69.2	2	15.4	0.09	13	50.0	13	50.0	0.08
Mother	17	46.0	18	48.6	2	5.4	0.59	52	70.3	22	29.7	0.39
Father	11	37.9	15	51.7	3	10.4	0.91	37	63.8	21	36.2	0.73
Sibling	22	37.3	34	57.6	3	5.1	0.19	78	66.1	40	33.9	0.95
Uncle/aunt	32	48.5	28	42.4	6	9.1	0.44	92	69.7	40	30.3	0.29
First cousin	44	41.9	47	44.8	14	13.3	0.33	135	64.3	75	35.7	0.56
	**Genotype frequencies**						***P*^b^**	**Allele frequencies**				***P*^b^**
***MTHFR* 677**	**C/C**		**C/T**		**T/T**			**C**		**T**		
	***N***	**%**	***N***	**%**	***N***	**%**		***N***	**%**	***N***	**%**	
Other rel.	6	46.2	5	38.5	2	15.3	0.60	17	65.4	9	34.6	0.96
1st degree relatives	52	40.9	67	52.8	8	6.3	0.14	171	67.3	83	32.7	0.72
2nd degree relatives	34	48.6	30	42.9	6	8.5	0.53	98	70.0	42	30.0	0.32
3rd degree or higher relatives	46	41.1	50	44.6	16	14.3	0.12	142	63.4	82	36.6	0.22
Paternal line	33	50.8	26	40.0	6	9.2	0.38	92	70.8	38	29.2	0.16
Maternal line	47	40.9	52	45.2	16	13.9		146	63.5	84	36.5	
***SLC19A1***	**A/A**		**A/G**		**G/G**			**A**		**G**		
Affected individual	4	30.8	7	53.9	2	15.3	0.32	15	57.7	11	42.3	0.15
Mother	7	18.9	19	51.4	11	29.7	0.99	33	44.6	41	55.4	0.93
Father	7	24.1	16	55.2	6	20.7	0.45	30	51.7	28	48.3	0.22
Sibling	11	18.6	35	59.4	13	22.0	0.34	57	48.3	61	51.7	0.31
Uncle/aunt	11	16.7	27	40.9	28	**42.4**	**0.04**	49	37.1	83	62.9	0.07
First cousin	16	15.2	56	53.4	33	31.4	0.66	88	41.9	122	58.1	0.44
Other rel.	2	15.4	8	61.5	3	23.1	0.93	12	46.2	14	53.8	0.83
1st degree relatives	25	19.7	72	56.7	30	23.6	0.09	122	48.0	132	52.0	0.06
2nd degree relatives	11	15.7	30	42.9	29	41.4	0.07	52	37.1	88	62.9	0.08
3rd degree or higher relatives	18	16.0	59	52.7	35	31.3	0.88	95	42.4	129	57.6	0.67
Paternal line	12	18.5	34	52.3	19	29.2	0.40	58	44.6	72	55.4	0.21
Maternal line	17	14.8	53	46.1	45	39.1		87	37.8	143	62.2	

a *Paternal line consists of uncles and aunts, grandparents, first cousins and first cousins once removed who are related to the affected individual through the father; maternal line consists of uncles and aunts, grandparents, first cousins and first cousins once removed who are related to the affected individual through the mother*.

b *For specific relatives, each category was compared with all other relatives to calculate P-values. For example, fathers were compared with all other relatives to calculate the P-value. For degree of relationship to the affected individual, each category was compared with a combination of the other two to calculate the P-value. For example, 1st degree relatives were compared with 2nd and 3rd degree relatives combined to calculate the P-value. P-values were calculated using a chi-square test unless one or more cells contained less than 5, in which case Fisher’s exact test was used*.

Relatives of individuals with NTDs might be expected to have genotypes associated with increased or decreased risk for NTDs, and the prevalence of these genotypes could increase or decrease with degree of closeness to the affected individual. For *MTHFR* 1298A>C, a lower percentage of first-degree relatives (34.6%) had the higher folate-metabolizing AA genotype compared with second-degree (50.0%) relatives and third-degree and higher (53.6%) relatives (*p* = 0.009). Similarly, the A allele was less common in first-degree relatives (61.4%) than in second-degree relatives (70.0%) and third-degree and higher (73.7%) relatives (*p* = 0.005). When the genotype (AA vs. A/C, C/C) and relatives (first-degree relatives vs. all other relatives) were dichotomized, the odds ratio for the association of the AA genotype with first-degree relatives was 0.49 (0.30–0.78, *p* = 0.002) (data not shown). Consistent with this, a lower percentage of siblings, 30.5%, had the AA genotype compared to 48.3% among other relatives (*p* = 0.04, Table 4). We did not observe a consistent trend of genotypes being more or less common in relatives more closely related to the affected individual for any of the other genotypes (Table 4).

We observed some associations with individual categories of relatives. For example, fewer first cousins had the higher folate-metabolizing *DHFR* del/del genotype compared with all other relatives (12.4 vs. 22.1%, *p* = 0.11, Table 4 and data not shown), the higher folate-metabolizing *MTHFD1* GG genotype was higher in uncles and aunts compared with all other relatives (51.5 vs. 31.6%, *p* = 0.003) (Table 4), and the lower folate-metabolizing *SLC19A1* GG genotype was more common in uncles and aunts than in other relatives (42.4 vs. 26.6%, *p* = 0.04) (Table 4). However, the meaning of these isolated associations is unclear.

### Number of risk genotypes

While the frequencies of individual genotypes did not differ significantly between maternal and paternal relatives (Table 4), the number of risk genotypes, defined as the number of homozygous genotypes associated with lower folate metabolism, did show significant variation between the maternal and paternal lines (*p* = 0.02) (Table 5). A lower percentage of maternal relatives (20.9%) had no (0) risk genotypes compared with paternal relatives (41.5%), while more maternal relatives (54.8%) than paternal relatives (35.4%) had 1 risk genotype. When the risk genotypes were dichotomized (0, 1–3), the odds ratio for association with the maternal line compared with the paternal line was 2.69 (1.38–5.25, *p* = 0.004).

**Table 5:** **Number of risk genotypes by relationship to affected individuals with NTDs and lineage, northeast region of the Republic of Ireland and Northern Ireland, 1995–2009**.

	**Number of risk genotypes/low metabolizer genotypes**								***P*-value^a^ (Fisher’s exact test)**
	**0**		**1**		**2**		**3**		
	***N***	**%**	***N***	**%**	***N***	**%**	***N***	**%**	
Maternal line	24	20.9	63	54.8	24	20.9	4	3.4	0.02
Paternal line	27	41.5	23	35.4	12	18.5	3	4.6	
Affected individual or mother	22	44.0	11	22.0	15	30.0	2	4.0	0.004
All other relatives	86	31.6	130	47.8	47	17.3	9	3.3	
Mother	17	46.0	7	18.9	12	32.4	1	2.7	0.005
All other relatives	91	31.9	134	47.1	50	17.5	10	3.5	

a *P-values compare maternal vs. paternal line, affected individual or mother vs. all other relatives, and mother vs. all other relatives*.

The numbers of risk genotypes in affected individuals and mothers (*p* = 0.004) or mothers alone (*p* = 0.005), compared with all other relatives were also statistically significant (Table 5). A higher percentage of affected individuals and mothers (30.0 vs. 17.3%) and mothers alone (32.4 vs. 17.5%) had 2 risk genotypes compared with all other relatives, while a lower percentage of affected individuals and mothers (22.0 vs. 47.8%) and mothers alone (18.9 vs. 47.1%) had 1 risk genotype. A higher percentage of affected individuals and mothers (44.0 vs. 31.6%) and mothers alone (46.0 vs. 31.9%) had 0 risk genotypes.

## Discussion

In the Irish families in our study, we found that maternal relatives were more likely than paternal relatives to have one or more genotypes associated with decreased folate metabolism. As expected from previous studies, mothers and affected individuals had differential distributions of risk genotypes compared with all other relatives.

The observed increased number of affected relatives in the maternal lineage of families could be due to genetic and/or epigenetic mechanisms. A simple genetic explanation would be that more maternal relatives have alleles associated with increased risk for NTDs simply due to chance, so the likelihood of inheriting these alleles from the maternal side would be increased. With a simple genetic model, we would expect that relatives would share the same risk genotype as the affected individuals and/or mothers. However, we did not observe this, but instead found that maternal relatives had an over-abundance of any risk genotype, rather than one specific genotype. We found that more maternal relatives had one risk genotype, while more paternal relatives had no risk genotypes. However, if this were the main explanation behind the increased number of affected individuals on the maternal side, we would expect that relatives more closely related to the affected individuals would share more genetic risk factors, and thus the prevalence of higher risk genotypes would be greater in first-degree relatives than in more distant ones. However, with the exception of *MTHFR* 1298, we did not observe consistent patterns of increasing numbers of risk genotypes with closer degree of relationship to the affected individual.

The mother's genotype during pregnancy plays a significant role in NTD risk, best studied in *MTHFR* (Yan et al., 2012), and the inheritance of these alleles would necessarily come from the maternal line. In this case, mothers and possibly affected individuals might be expected to have an increased prevalence of genotypes associated with greater NTD risk. In our study, fewer affected individuals had the higher folate-metabolizing *MTHFR 677* CC genotype, consistent with earlier reports that the homozygous TT genotype is a risk factor for NTDs (Shields et al., 1999; Botto and Yang, 2000). Likewise, affected individuals and mothers, as well as mothers only, were more likely to have 2 risk genotypes. Our study of these associations was limited by our small sample size and was not the main objective of this study. While our findings were consistent with previous literature, these associations alone were not strong enough to explain the increased number of affected relatives on the mother's side, suggesting that other mechanisms are also at work.

Whenever there is preferential inheritance of a condition from the maternal vs. paternal line, epigenetic effects are strongly suspected. The genes included in our study are not located in known imprinted regions. However, folate metabolism is critical to the production of one-carbon subunits needed for epigenetic modification. Certain variants of folate metabolism genes (possibly including those in our study), which are associated with NTD risk, might affect proper imprinting and establishment or maintenance of other epigenetic changes. These epigenetic effects could occur in the maternal genome and would have been initiated when the mother was a fetus herself, but may also have taken place in the genomes of the mother's parents (Crider et al., 2012). If this mechanism were at work, the maternal and paternal grandparents' genotypes would be of importance. Unfortunately, we were unable to obtain samples from many grandparents. However, aunts, uncles, and first cousins provide an indirect measure of the grandparents' genotypes, and significant associations for these relatives were seen for *DHFR*, *MTHFD1*, and *SLC19A1*. The increased number of risk genotypes in maternal vs. paternal relatives is consistent with an epigenetic mechanism.

Our study is the first to look at the genotypes of second- and third-degree or more relatives in families affected by NTDs and to begin to explore the genetic and epigenetic mechanisms behind the increased NTD and overall birth defects risks in these families. However, our study is preliminary and has a number of limitations. The limitations of our study include a relatively small sample size, lack of triads (both parents and offspring) among distant relatives to allow for heritability testing, and inadequate number of samples from grandparents to evaluate epigenetic effects. Additionally, more maternal than paternal relatives participated in the study. However, while this might have affected the stability of the point estimate for the association of risk genotypes with the maternal vs. paternal line, it did not affect the magnitude or direction. The response rate was relatively low for our study, and the participation of families and of relatives within families could be biased and not reflect the true underlying distribution of families with NTDs in Ireland. However, selection for and participation in the study was independent of genotype and thus participants can be considered close to a random sample of relatives. The candidate gene approach used here evaluated a small number of polymorphisms and would have missed associations with other variants in folate metabolism pathway genes. An important limitation of our study (and others) is that we were not able to measure the mother's intake of folate and folic acid during pregnancy. The genes included in this study function in folate metabolism, so that their effect depends on the levels of folate present in the body. For example, high intake of folic acid and folate could mask genetic effects that would be seen at lower intake levels. Lastly, the number of multiple comparisons evaluated in our study likely produced some spurious findings.

Our results are consistent with both genetic and epigenetic mechanisms contributing to the increased NTD and overall birth defects risk in the maternal lineage. The diversity of genetic risk seen in these families suggests that etiology of NTDs is multi-faceted. Future studies will benefit from including other relatives, especially grandparents, in addition to mothers, fathers, and affected individuals, especially if epigenetic mechanisms are to be addressed.

### Conflict of interest statement

The authors declare that the research was conducted in the absence of any commercial or financial relationships that could be construed as a potential conflict of interest.
